# Nanoplasmonic sensors for extracellular vesicles and bacterial membrane vesicles

**DOI:** 10.1186/s40580-024-00431-8

**Published:** 2024-06-25

**Authors:** Aparna Neettiyath, Kyungwha Chung, Wenpeng Liu, Luke P. Lee

**Affiliations:** 1 Renal Division and Division of Engineering in Medicine, Department of Medicine, Brigham and Women’s Hospital, Boston, MA 02115 USA; 2 Harvard Medical School, Harvard University, Boston, MA 02115 USA; 3 Department of Bioengineering, University of California, Berkeley, CA 94720, USA; 4 Department of Electrical Engineering and Computer Science, University of California, Berkeley, CA 94720, USA; 5 Institute of Quantum Biophysics, Department of Biophysics, Sungkyunkwan University, Suwon 16419, Korea; 6 Department of Chemistry and Nano Science, Ewha Womans University, Seoul 03760, Korea

**Keywords:** Extracellular vesicle, Bacterial membrane vesicle, Nanoplasmonics, EV sensing, Optical sensor, Label-free sensor, Surface-enhanced Raman spectroscopy (SERS), Multiplexed detection, Point-of-care diagnostics, Environmental sensors, Monitoring sustainability

## Abstract

Extracellular vesicles (EVs) are promising tools for the early diagnosis of diseases, and bacterial membrane vesicles (MVs) are especially important in health and environment monitoring. However, detecting EVs or bacterial MVs presents significant challenges for the clinical translation of EV-based diagnostics. In this Review, we provide a comprehensive discussion on the basics of nanoplasmonic sensing and emphasize recent developments in nanoplasmonics-based optical sensors to effectively identify EVs or bacterial MVs. We explore various nanoplasmonic sensors tailored for EV or bacterial MV detection, emphasizing the application of localized surface plasmon resonance through gold nanoparticles and their multimers. Additionally, we highlight advanced EV detection techniques based on surface plasmon polaritons using plasmonic thin film and nanopatterned structures. Furthermore, we evaluate the improved detection capability of surface-enhanced Raman spectroscopy in identifying and classifying these vesicles, aided by plasmonic nanostructures. Nanoplasmonic sensing techniques have remarkable precision and sensitivity, making them a potential tool for accurate EV detection in clinical applications, facilitating point-of-care molecular diagnostics. Finally, we summarize the challenges associated with nanoplasmonic EV or bacterial MV sensors and offer insights into potential future directions for this evolving field.

**Graphical Abstract:**

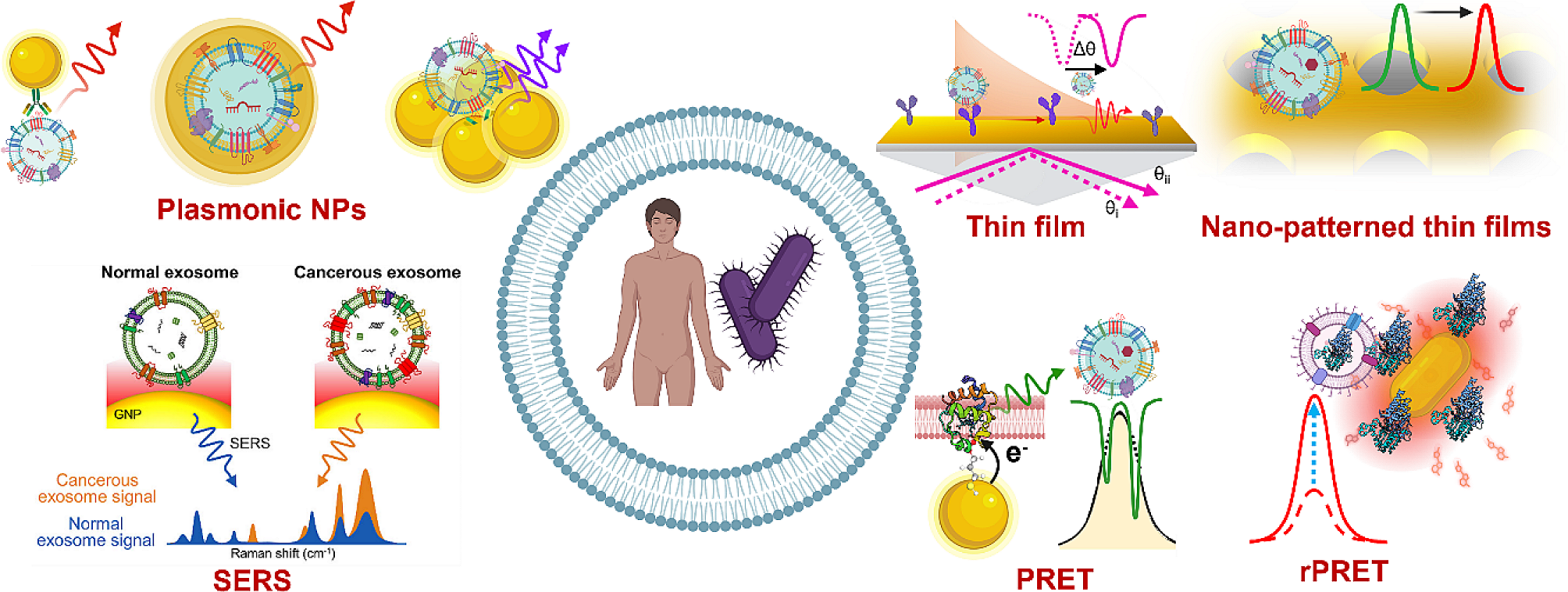

## Introduction

Cells secrete extracellular vesicles (EVs) to mediate cell-cell communication. These also include bacterial membrane vesicles (MVs) or bacterial outer membrane vesicles (OMVs) released by bacteria. Because of the biogenesis of these vesicles [1], their contents (i.e., DNAs, RNAs, proteins, lipids, enzymes) give us a snapshot of the cell life by reflecting the intracellular composition of the cell origin and their communications. EVs are critical intercellular communicators [1, 2] in the pathogenesis of many diseases, such as neurodegenerative diseases and cancers. Bacterial MVs mediate bacterial communication and have diverse functions [3]. Since molecular cargo carried by body-cell-derived EVs can reflect disease status, they are promising biomarkers as essential biosignatures for early disease diagnosis [4] and restorative therapy. Bacterial MVs can give information about health and disease [5] while also being useful for environmental monitoring [6, 7]. This review discusses advances in the nanoplasmonic sensing of these vesicles.

There have been several bottlenecks in the clinical translation of EVs or MVs as biomarkers because of the slow isolation of these vesicles and the need for more sensitive and selective detection techniques. While the traditional isolation methods of EVs or MVs, such as differential ultracentrifugation [8, 9], size-based isolation [10, 11], and polymer-based precipitation [12, 13], are being utilized well, a recent breakthrough in EV isolation using an advanced ultrafiltration technique known as EXODUS, has enabled ultrafast EV isolation [14]. To fully harness the potential of EVs for diagnosis and therapy, there is a pressing demand for next-generation EV sensing technology to complement the advancement in EV isolation. Enzyme-linked immunosorbent assay (ELISA) and Western blotting methods commonly used to detect EV surface biomarkers have low sensitivity and cannot be used with low sample volumes [15]. Nanoplasmonic biosensors have provided extreme sensitivity and efficiency in frontier areas of translational medicine [16–18]. Their adaptability and potential for facilitating high-throughput detection make nanoplasmonic sensors particularly suitable in EV-based diagnostics. Therefore, this article aims to review current research in nanoplasmonic EV sensing, shedding light on its accomplishments, challenges, and future directions.

The distinct structural and biochemical properties of EVs have been studied over the years. EVs are of endocytic origin and are released to extracellular space upon the fusion of plasma membrane and multivesicular bodies. EVs exhibit unique protein signatures, including those involved in membrane transport and fusion, heat shock proteins, and tetraspanins on the vesicle membrane, such as CD63, CD9, and CD81. Moreover, EVs carry diverse genetic materials, including DNAs, RNAs, mRNAs, miRNAs, and circRNAs. The concentration and characteristic features of EVs are altered in various pathological conditions, including cancers [15, 19], neurodegenerative diseases [20], and cardiovascular diseases [21]. Due to their ability to cross the blood-brain barrier while carrying genetic cargo, EVs make it easy to obtain information about nerve and brain function using them [22]. Also, they can be found in several types of biological fluids, such as saliva [23], blood serum [24], urine [25], cerebrospinal fluid [26], breast milk [27], and tears [28]. Thus, EVs can be a noninvasive medical diagnostic tool for disease detection, prognosis, and treatment monitoring [29]. This liquid biopsy approach offers the advantage of avoiding the pain and complications associated with traditional tissue biopsies.

When it comes to bacterial MVs, they are typically 40–400 nm in size and carry specific cargo, including enzymes, DNAs, RNAs, endolysins, toxins, proteins, phages, and other molecules [30]. Most bacteria produce MVs, and apart from communication, they have diverse functions such as DNA transfer, bacteriophage interception, and cell detoxification [3]. Bacterial MVs can indicate diseases and human health; for example, bacterial MVs from gut bacteria can indicate gut diseases and be collected from biofluids such as urine and blood [5]. Gut microbiota influences bidirectional interactions in the gut-brain axis, which links brain and intestinal functions [31]. Changes in brain-gut-microbiome communications can be connected to the pathogenesis of diseases such as irritable bowel syndrome, obesity, depression, anxiety, and neurological disorders [32–34]. Hence, monitoring bacterial MVs released by gut microbiota can indicate these diseases. One example is the gut bacteria A. muciniphila and its MVs, which are involved in serotonin signaling and metabolism through the gut-brain axis [35]. Isolation and detection of MVs, in this case, can identify serotonin-related disorders and also provide a new therapeutic strategy. Similarly, monitoring the MVs from cyanobacteria, such as Prochlorococcus [6], can be a way to estimate environmental health. Environmental variables that these phytoplankton encounter affect the amount and structure of the MVs from them, and monitoring MVs can indicate environmental toxins and viral infections. In an ecosystem like the ocean, MVs constitute only a tiny fraction of colloidal particles per ml in seawater [7], posing a bottleneck for bacterial MV-based environment monitoring and hence need enrichment and isolation similar to EVs.

Excellent sensitivity and specificity when detecting biomolecules are expected from a biosensor. The COVID-19 pandemic has reinforced the importance of point-of-care (POC) diagnostics in our society. While many such diagnostic kits rely on chemical analysis, testing kits that can be used at home can also be made using nanoplasmonic materials by converting adsorption or desorption events of the analytes into optical signals. This transduction is achieved through the resonant interaction between the oscillating surface electrons of the metallic nanostructures and the incident light. Their resonant conditions are highly susceptible to the refractive index (RI) change occurring due to the adsorption or desorption events on the surface, which nanoplasmonic sensors can efficiently capture.

Nanoplasmonics-based biosensors can be categorized into three primary types: nanoparticle-based localized surface plasmon resonance (LSPR) sensing, thin film- or nanopattern-based SPR sensing, and surface-enhanced Raman spectroscopy (SERS) sensing. The sensitivity (*S*) of LSPR and SPR sensors can be expressed in terms of refractive index sensitivity (*S*_*RI*_), which describes the sensitivity to the change in refractive index upon the binding event of analytes to the sensor surface and the efficiency of binding of analytes (*E*_*b*_) at a concentration $c:S=({\partial}P/{\partial}n)\cdot({\partial}n/{\partial}c)=S_{RI}\cdot{E_{b}}$.

*P* represents output signals, which could be the resonance wavelength (*λ*) for LSPR sensors or the resonant angle (*θ*) for SPR sensors, and *n* denotes the RI. *E*_*b*_ is mainly determined by the binding affinity between the biorecognition element and the analyte, as well as the efficiency of surface functionalization. Enhanced *S*_*RI*_ can be accomplished by increasing the magnitude of the electric field on the sensing surface. A higher electric field on the surface of plasmonic materials induces a larger change in *P*, as the probability of probing analyte binding within the evanescent field increases [36].

In the case of SERS, its performance is primarily influenced by the local electric field (*E*_*loc*_) mechanism, which can be described by the enhancement factor (EF) with incident light having electric field strength *E*_0_ $EF\approx {\left|{{E}_{loc}}/{{E}_{0}}\right|}^{4}$.

The sizes of EVs and MVs are well-matched within the extension of the local electric field of a nanoplasmonic sensing element (∼ 200 nm), making them capable of perturbing the field and generating a signal response upon contact with the sensor. Unlike other biomarkers that are smaller in size, EVs can perturb the field significantly, producing excellent signals from a nanoplasmonic sensor. While nanoplasmonic techniques offer versatility in sensing a wide range of biomolecules [38–40], the design of EV and MV sensors necessitates a specific focus on capturing EVs. As EVs share vesicular characteristics with viruses, immunocapturing using various surface proteins is possible. *E*_*b*_ can be optimized through proper surface functionalization techniques, improving the overall performance of nanoplasmonic EV and MV sensors.

While plasmonic biosensors are used to detect a wide range of biomarkers, the detection of EV and MV biomarkers holds particular opportunities for disease diagnosis, prognosis, and therapy. These biomarkers, ubiquitous in all biofluids, contain encapsulated information and exhibit larger sizes compared to other molecules. The inherent biocompatibility of EVs and MVs in treatment makes them impactful biomarkers for nanoplasmonic sensing techniques. Moreover, nanoplasmonic biosensors for EVs and MVs offer several advantages, such as high sensitivity, non-invasiveness, and biocompatibility. Their tunability in shape and size enables the production of diverse output signals, while the capability for multiplexing detection allows for the simultaneous sensing of multiple biomarkers. Also, plasmonic materials are very suitable for functionalization with capture molecules, ensuring the specific detection of targeted biomarkers. In addition, nanoplasmonic techniques provide the feasibility of designing compact and portable devices with the integration of microfluidics, making them ideal for POC applications.

We will discuss the fundamentals of different nanoplasmonic sensing methods and explore their applications in EV sensing. Notably, gold nanoparticles, nanoparticle multimers, and various nanostructured plasmonic thin films exhibit significant capabilities in sensing EVs. We will also emphasize nanoplasmonic bacterial MV detection. Furthermore, we will highlight the unique potential of SERS for EV surface biomarker detection, offering a valuable tool in this field. We will emphasize the importance of a tailored design to effectively capture and analyze EVs, harnessing the potential of nanoplasmonics for advanced diagnostics.

## Plasmonic nanoparticles for EV sensing

**Fig. 1:**
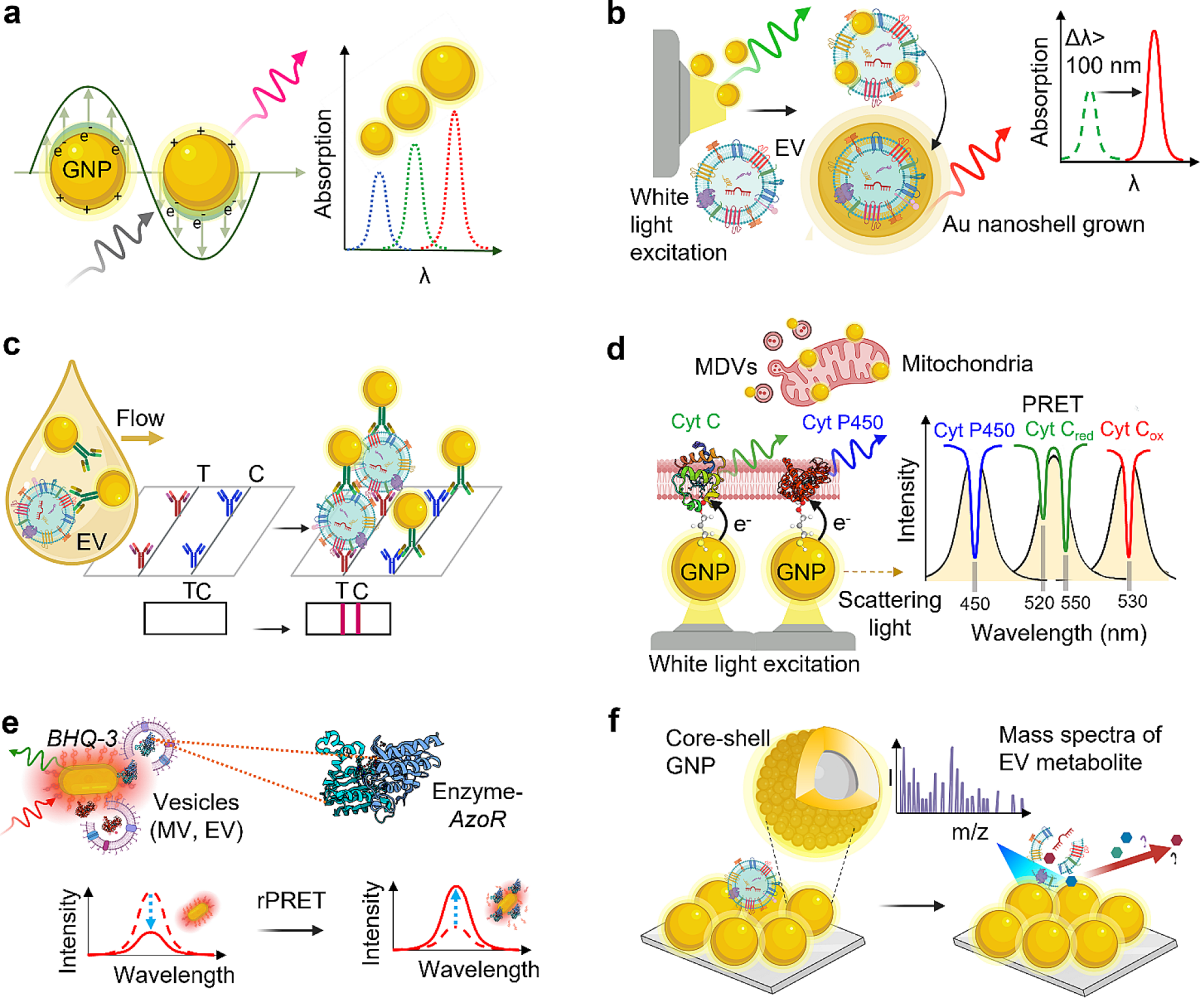
Plasmonic nanoparticles for EV sensing based on change in size, scattering intensity, and energy transfer - **a**, Localized surface plasmon resonance (LSPR) of plasmonic gold nanoparticle (GNP) and size-dependent shift in light absorption. **b**, In-situ growth of Au nanoshell on EV, causing a significant change in GNP diameter and > 100 nm change in absorption wavelength that can be visually detected [42]. **c**, GNP labels for immunosandwich EV detection with antibodies in lateral flow immunoassay detection based on scattering intensity [43]. **d**, GNP labels for Cyt C and Cyt P450 marking on mitochondria, plasmonic resonance energy transfer (PRET) enables spectroscopic imaging in situ [44]. **e**, Plasmonic nanoantennas to monitor dynamic intercellular communications across distances by reversed plasmonic resonance energy transfer (rPRET), which is achieved by interfacing with resonating black hole quencher (BHQ-3) molecules for highly selective and sensitive detection of released enzymes such as azoreductase that being released via EVs or MVs [45]. **f**, Core-shell GNPs enhance the mass spectrum of EV metabolites [46]. The cartoons for enzyme molecules in **d** and **e** were created from the RCSB protein data bank [47–49]

A nanoplasmonic antenna consisting of metallic nanoparticles shows enhanced light-matter interaction compared to the nanoparticle of dielectric materials having similar diameters [41] (Fig. 1a). LSPR occurs when electron dipole-induced collective oscillation of the electron cloud at the surface of a metallic nanoparticle (NP) resonates with the light of specific frequency, causing amplified and confined electric field. This plasmon energy is dissipated by scattering, depending on particle size and outer media. Depending upon the size of the NP, the oscillation frequency of the electron cloud also varies. Hence, with the increasing diameter of NP, a red shift in the light absorption spectrum can be observed (Fig. 1a). The resonant frequency shifts upon the change in the refractive index of the media. Moreover, unlike traditional coloring materials such as quantum dots, fluorescent organic dyes, pigments, and green fluorescent protein (GFP) that suffer from photobleaching, plasmonic NPs exhibit highly stable chemical and physical properties. These aspects make nanoplasmonic antenna an attractive photobleaching-free label and optical sensor platform for EV detection.

The growth of an EV-templated nanoplasmonic shell is an effective strategy that leads to a remarkable color change and higher detection sensitivity [42]. As indicated in Fig. 1b, GNPs with a diameter of ∼ 9 nm readily adsorb onto the phospholipid bilayer of EV via electrostatic forces, effectively covering the surface of the EVs. Using these EV-GNPs as a template, an in-situ growth process of the Au shell creates an outer gold nanoshell around the template. The resulting nanoplasmonic structure is larger than the initial template and GNPs, leading to a substantial shift in LSPR absorbance wavelength, exceeding 100 nm. This significant spectral change could be easily detected using a smartphone camera. The claimed sensitivity is 1000 times better than that of ELISA, with a detection limit of 1500 EVs, and it showed higher accuracy in classifying cancer (area under the curve (AUC) of 0.97). Nanoplasmonic antennas are significant in analyzing EV biomarkers, offering fast, wash-free, and empathetic capabilities for clinical trials and parallel processing [42].

Lateral flow immunoassay (LFIA) is a low-cost POC biomarker sensing platform [50] similar to ELISA, but without the multi-step process, making it suitable for EV sensing. Owing to their optical properties, gold nanoparticles are proven to be excellent labels in LFIA [51]. A gold nanoparticle (GNP)-labeled LFIA EV sensor is shown in Fig. 1c. Here, EVs in a biofluid, flowing by capillary action through control and test immune-sandwich structures, interact with specific anti-CD63 or CD81 antibodies. EVs in the biofluid need to be labelled with antibody-conjugated GNPs to enable their capture and further colorimetric detection. The GNP-labelled LFIA technique achieved a detection limit of 8.5 × 10^5^ EVs per µl [43]. Multiplexed detection of analytes is also possible in LFIA by adding spatially separate sequential detection sites employing different target antibodies on a single strip [43, 52]. The small size of GNP labels (< 50 nm) allows them to be closely packed on the test line [53]. Secondary labels such as plasmonic NPs [54] or enzymes [55] can further enhance the optical signal, thereby improving the sensitivity of naked eye detection.

Besides refractive index-dependent LSPR sensing, plasmonic resonance energy transfer (PRET) [56, 57] has been explored in spectroscopic imaging of living cells in a non-destructive and label-free manner [44]. Plasmonic resonance energy can be transmitted from plasmonic components to emitting materials nearby, detected by measuring the scattering spectrum of their combination. This spectrum often exhibits one or a few dips, corresponding to the absorption peaks of the emitting materials or an overall decrease in intensity, depending on the coupled systems. These dips in the scattering spectrum indicate the adjacent emitting material’s absorption of plasmonic resonance energy. Figure 1d illustrates how GNPs on the mitochondrial membrane act as nanoplasmonic antennas, enabling the monitoring of Cyt C released from the mitochondria and their oxidation state by capturing energy transfer at the junction between the mitochondrial membrane and GNP.  A similar approach using PRET can detect molecules such as Cytochrome P450 (Cyt P450) in the mitochondria as well, as shown (Fig. 1d). Cyt C plays critical roles in metabolic activities, cell respiration, apoptosis, and regulation of several diseases [58]. However, monitoring Cyt C in situ has been considered challenging due to the limitations of conventional analytical tools, such as flow cytometry [59], Western blot [60], ELISA [61], and electrochemical biosensing [62]. Real-time quantum biological electron tunneling (QBET) is formed in the presence of plasmonic NP with Cyt C, through which quantum electron tunneling and energy transfer happen across the interfacial molecular layer. The size of GNP is selected to match Cyt C absorption wavelength with the GNP scattering. Upon plasmon energy transfer to Cyt C via PRET, the resultant spectrum exhibits characteristic dips indicating the presence of Cyt C. A minimum concentration of 1 µM of Cyt C was distinguished by QBET signal. PRET-based spectroscopic detection of Cyt C can be repurposed for detecting mitochondria-derived vesicles (MDV) [63, 64]. This will provide spatial information with oxidation state simultaneously, helping to understand the mitochondrial functions and the involvement of EVs in them [63].

Plasmonic nanoantennas have enabled the monitoring of dynamic intercellular communications across distances through reversed plasmonic resonance energy transfer (rPRET). This is achieved by interfacing with resonating black hole quencher molecules, which enables highly selective and sensitive detection of the enzyme azoreductase.(Fig. 1e) [45]. This is especially important in the case of bacterial MVs, which carry specific cargos and represent bacterial secretion pathways [30, 65]. Plasmonic nanoantenna can be employed to detect enzymes in bacterial MVs and EVs. Bacterial MVs represent bacterial pathogenesis, thereby enlightening people about health and disease [5]. Also, bacterial MVs can be an indicator for environmental monitoring, including in ocean and marine ecosystems [6, 7]. Figure 1e indicates a notable bacterial MV sensor using a gold nanorod (GNR) as a nanoplasmonic antenna. The gold nanorod (GNR) surface is modified with black hole quencher (BHQ-3) molecules, which quenches the scattering of GNR. These GNR/BHQ-3 nanoantenna serve as indicators for bacterial MVs carrying enzymes such as azoreductase (AzoR). As AzoR induces degradation of BHQ-3, the suppressed intensity of GNR scattering is restored, in a reverse process of plasmonic resonant energy transfer, termed as rPRET. The rPRET technique can detect the AzoR enzyme down to a detection limit of 5 nM. Also, enzymes of MVs can be detected up to 3 μm distance from the bacteria (such as *E*. *coli*) that secretes the vesicle. This concept of plasmonic nanoantenna can also be explored for detecting and monitoring metabolic enzymes. For instance, it can be applied to label-free direct detection of enzymes of diagnostic significance, an indicator for drug abuse and the presence of neurotoxin [66] packaged within EVs.

Plasmonic nanomaterials have proven to be valuable when combined with other analytical tools. For instance, the utilization of silica core-gold shell NPs (Fig. 1f), fabricated by Au sputtering, has demonstrated their utility in enhancing the performance of mass spectrometry analysis of EV metabolites [46]. Mass spectrometry (MS) is a high-throughput technique that can provide dense information regarding various small molecular biomarkers present in EVs [67]. Signal enhancement in MS is achieved by enhanced ionization upon LSPR coupling [68]. Additionally, plasmonic substrate-assisted photothermal heating has enhanced MS signals from EVs [69]. Early indications of diseases, such as non-small cell lung cancer (NSCLC), can be identified from EV metabolite biomarkers [46]. It is notable that metabolic fingerprints could be detected from very small volumes of EVs, with as little as 400 pL being sufficient for detection.

Another notable sensing method is a nanoplasmonic-enhanced scattering assay used to detect two EV protein biomarkers simultaneously using GNPs of two different geometries as labels [70]. In this case, dark-field microscope imaging detects the nanoplasmon-enhanced scattering. Nanoplasmonic immunoassays offer integrated EV diagnostic assays, making them particularly valuable with the possibility of integration with the microfluidic isolation step [71]. Additionally, the emerging techniques of ultrafiltration-based rapid isolation methods enable the collection of EVs from smaller volumes of biofluids (blood [72], tear [73], saliva [14]) to be analyzed while reducing diagnosis time and laboratory costs without external labeling.

**Fig. 2:**
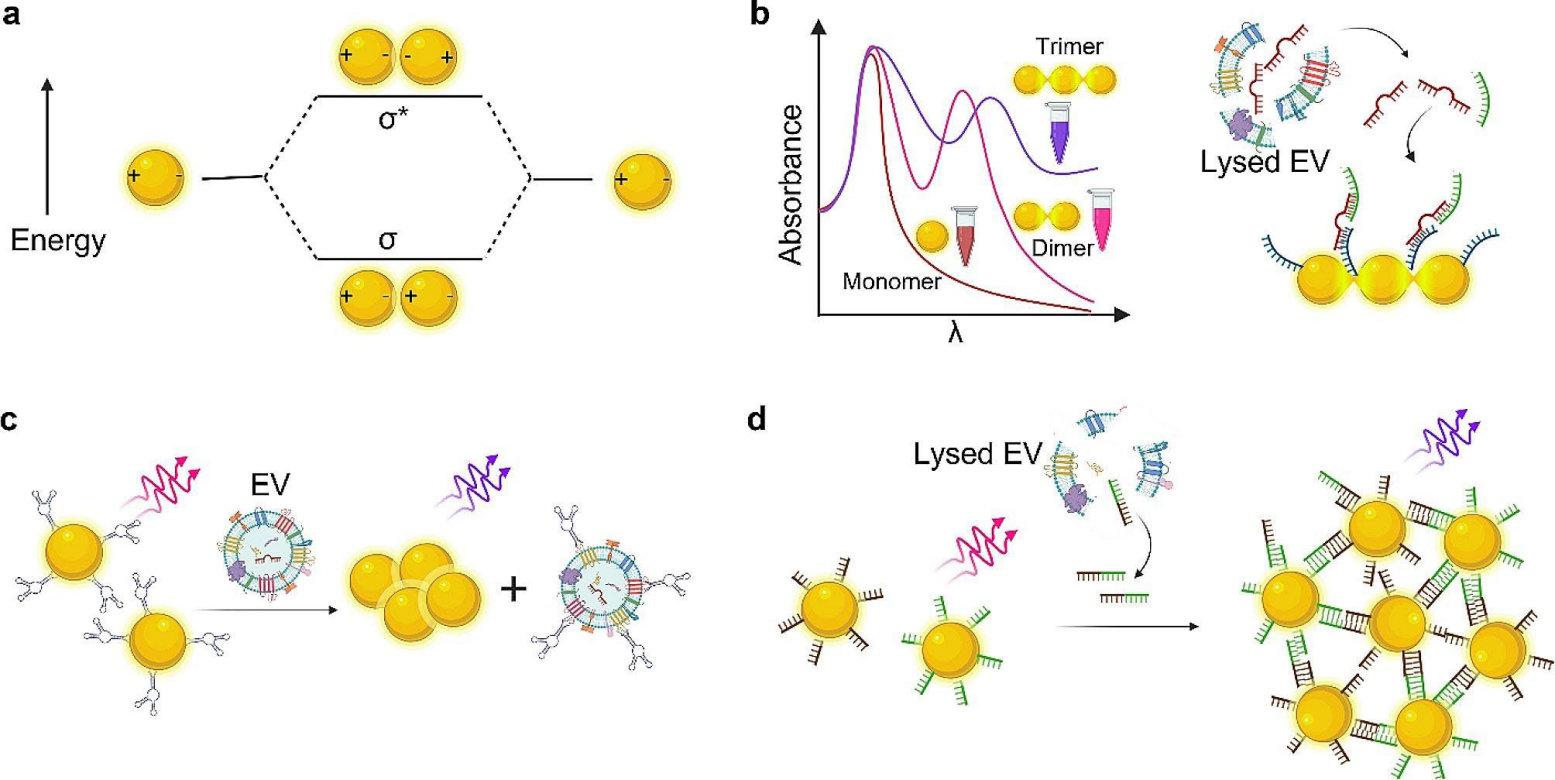
GNP multimer-based EV sensing. **a**, GNP dimer and plasmonic hybridization, causing a change in surface plasmon resonance energy. **b**, Absorbance of GNP-monomer, dimer, and trimer; the first LSPR peak due to transverse modes is identical for all the cases, whereas the second LSPR peak due to longitudinal modes appears in dimer and red-shifts in trimer [79]; Trimer nanogaps for ultralow detection of EV miRNA. **c**, Competing interaction between aptamer-GNPs (weak) and aptamer-EV protein (vital), creating colorimetric detection of EVs due to GNP aggregation [80]. **d**, miRNA-induced hybridization causing aggregation of GNPs, resulting in a color shift, repurposable for EV miRNA detection [81]

GNP aggregation-based colorimetry has been widely explored in biosensing as aggregated GNPs provide color change and enhanced scattering intensity and electric field [74, 75]. When two individual GNPs are combined as dimers, their gap plasmon mode can generate considerable electromagnetic field enhancement at the junctions/nanogaps [76]. The plasmon hybridization model, analogous to molecular orbital theory, helps understand the collective nature of GNP dimers and multimers. For instance, two individual plasmon modes in proximity interact and couple each other, forming bonding (𝜎) and antibonding (𝜎*) dimer plasmon modes through redistribution of charge density and electromagnetic fields between two constituents (Fig. 2a). The bonding mode corresponds to the constructive interaction of plasmonic fields, resulting in a lower-energy mode (expressed by new peak at higher wavelength), while antibonding mode corresponds to the destructive interference, leading to a higher-energy mode. For large particle separations, the plasmon modes of GNP dimer follow classical dipole interaction, which varies slowly (1/distance^3^). In contrast, the interaction energy shifts for small particle separations are more substantial and vary fast [77]. GNP multimers show color change or shift in the spectrum, which can be explained by plasmon hybridization theory. Figure 2b shows the absorbance spectra of GNP-monomer, dimer, and trimer suspensions. In the case of GNP multimers, while the LSPR peak of monomeric GNP is retained, an additional LSPR peak evolves at a longer wavelength, corresponding to the bonding plasmon mode. GNP multimers have nanogaps with field confinements, where analyte molecules such as EV miRNA or EV proteins can be sandwiched (Fig. 2b) for high-sensitivity detection down to 10 aM [78], potentially enabling single molecule detection.

Aptamers are also used to capture EVs, in addition to antibodies. Aptamers, often called ‘chemical antibodies,’ are short nucleic acid oligomers known for their specific binding and excellent affinity to cellular proteins [82]. This characteristic makes them increasingly attractive for assays and sensors as an alternative to traditional antibodies. One intriguing nanoparticle-based sensor uses the competitive interaction between aptamer-bound GNPs and aptamer-bound EV proteins [80]. While the interaction between aptamers and GNPs is weak, the binding between aptamers and EV proteins is vital. In the presence of several EVs, GNPs are liberated from aptamers and aggregate with each other. This aggregation produces an LSPR shift (Fig. 2c). Furthermore, employing a panel of aptamers targeting ubiquitous EV surface proteins makes multiplexed colorimetric detection feasible. In another GNP aggregation-based sensor, RNA-modified GNPs detect miRNAs [81]. In this approach, modified GNPs are designed to hybridize with analyte miRNA, leading to the aggregation of GNPs and a consequent color change (Fig. 2d). Visual sensing of miRNA with a detection limit down to 5 nM is achieved. This technique holds promise for repurposable exosomal miRNA detection.

While it is possible to detect EVs with GNPs through simple LSPR-based RI sensing, the examples mentioned above have explored the advanced and straightforward nanoplasmonic systems by utilizing PRET or GNP multimer formation to maximize output signals. A comprehensive understanding of LSPR fundamentals is essential for developing advanced nanoparticle-based LSPR sensors with enhanced sensitivity, specifically for single EV sensing.

## Plasmonic thin film-based EV sensing

**Fig. 3:**
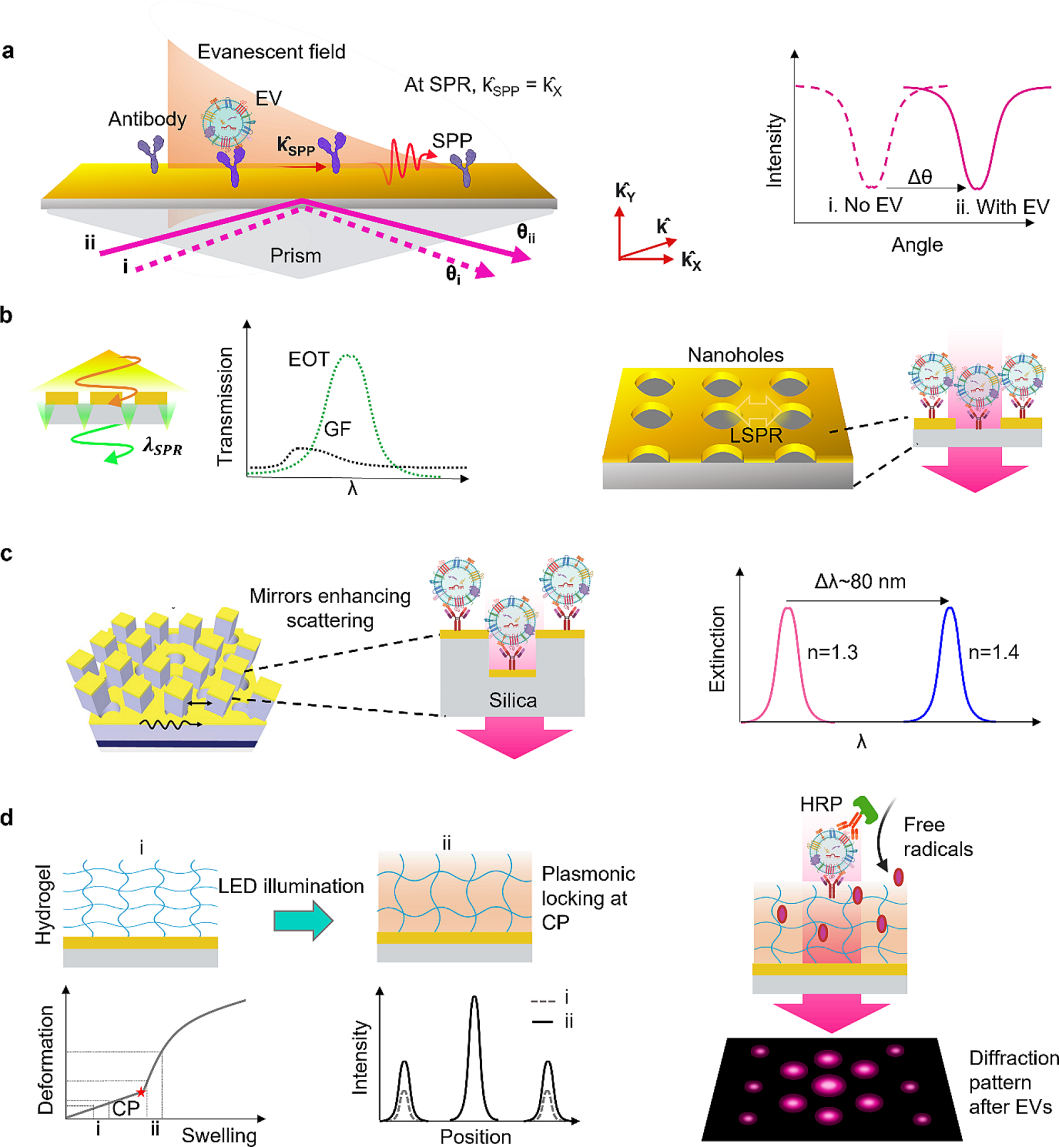
Plasmonic thin film-based EV sensing. **a**, Principle of surface plasmon resonance on a Gold film, showing evanescent wave, its extension (∼ 200 nm), and surface plasmon polariton (SPP) with wave vector k_SPP,_ EVs immunocaptured on plasmonic thin film using an antibody, measurable by SPR angle shift. **b**, Plasmonic nanoholes, transmitting light selectively due to LSPR and extraordinary optical transmission effect (EOT), EVs immunocaptured on plasmonic nanoholes, LSPR at the nanohole surface helps EV sensing [85]. **c**, Plasmonic mirrors enhancing scattering and providing significant LSPR spectral shift (∼ 80 nm), photonic plasmonic scattering for sensing immunocaptured EVs. Part of the figure reprinted with permission [90] Copyright 2018 Royal Chemical Society. **d**, Plasmonic locking of hydrogel metamaterial at the critical point (CP), which results in enhanced deformation per fixed change in swelling, immunocaptured EVs triggering the generation of free radicals by antibody-peroxidase activity, resulting in more crosslinking of hydrogel, and subsequent notable changes in the diffraction pattern [91]

On a plasmonic thin film, electron oscillations known as surface plasmons propagate along the substrate’s surface (${\hat k_X}$ in Fig. 3a). To excite propagating surface plasmon resonance, a prism or grating is used to couple the incident light with the electromagnetic surface waves called surface plasmon polaritons (SPP) (Fig. 3a). By matching the momentum of incident light with that of plasmon, surface plasmon resonance occurs and the direction of SPP (${\hat k_{SPP}}$) corresponds with the substrate surface direction (${\hat k_{SPP}}={\hat k_X}$). The evanescent field generated on the surface of the plasmonic substrate is perturbed by the analytes, including EVs, which adsorb or bind to the biorecognition elements on the surface. This leads to a change in RI, resulting in an alteration of the resonant condition of the substrate and consequently causing spectral or angular shifts. Since the decay length of the evanescent wave can be up to ∼ 200 nm, SPR spectroscopy is suitable for EV detection [83]. Plasmonic thin film substrates can be easily functionalized with a specific antibody through thiol chemistry to target specific EV surface proteins, such as CD63, to capture EVs (Fig. 3a). Subsequently, the SPR angle shift can be measured after injecting the analyte solution into the sensing chamber, allowing for qualitative and quantitative detection. In commercial SPR instruments (Biacore, GE Healthcare, BioNavis), the SPR angle shift is measured regarding RU (resonance units).

Advanced nanofabrication and lithography methods have integrated nanostructured plasmonic thin films into sensor substrates. The ability to create extreme scattering structures such as nanoholes and cavities has lowered detection limits [84]. EV detection can be conducted using SPR substrates with nanoholes with improved performance [85]. From a plasmonic substrate with a periodical nanohole structure, an unusual phenomenon known as extraordinary optical transmission (EOT) occurs with incident light of a specific wavelength (more significant than the array period), transmitted through the nanohole array unlike a gold film (GF) (Fig. 3b). This happens majorly due to the coupling of the incident light with the surface plasmons on the hole array, through which are transmitted by tunneling on the other side of the substrate [86]. Periodic plasmonic nanoholes can immunocapture EVs, and a conventional UV-vis spectrophotometer can measure transmission spectra with a peak shift upon the EV binding. This technique can provide four orders of magnitude higher sensitivity than traditional western blot [87] for EV protein detection and two orders higher than conventional ELISA [88]. Such substrates can be mass-produced and combined with imaging readouts to increase parallelism in detection. Moreover, strategic output amplification can be carried out by enzymatic deposition of insoluble products over the bound EVs over a plasmonic nanohole substrate [89]. This approach enabled superior sensitivity of about 200 exosomes and demonstrated EV sensing for diagnosing Alzheimer’s disease.

Combining localized and propagating surface plasmon resonance can be one approach to improve the sensitivity owing to the increased electric field. By incorporating a three-dimensional photonic crystal structure on a plasmonic mirror substrate featuring point-defect cavities, electric field enhancement, and high sensitivity can be achieved (Fig. 3c). Immunocapturing EVs on such a hybrid photonic crystal-plasmonic structure can result in a significant LSPR spectral shift due to the high sensitivity of nanostructured surface. The substrate enables a detection limit of 10^4^ EVs per ml with an extinction peak shift of 9 nm, displaying about 102 nm shift with a concentration of 10^11^ EVs per ml [90].

In another innovative approach, researchers have combined plasmonic thin film with a hydrogel-based mechanical metamaterial for EV sensing (Fig. 3d) [91]. This work involves a temperature- and redox-responsive hydrogel polymer that has been functionalized with antibodies and patterned onto Au thin film. When exposed to light, this configuration induces plasmonic heating, causing the hydrogel to undergo a plasmonic locking in a swollen state. The locked metamaterial exhibits much more significant deformation in response to a fixed amount of biomarker-induced swelling than an unlocked one. Upon immunocapturing EVs on the hydrogel metamaterial, antibody-horse radish peroxidase (HRP) activity produces free radicals that induce crosslinking in the hydrogel matrix, resulting in a chiral transformation of the hydrogel pattern. This notably amplifies the intensity of diffracted light, which can be easily detected with a smartphone camera. This hydrogel metamaterial on a plasmonic film demonstrates improved sensing performance, achieving sensitivity 10^3^ higher than that of standard ELISA, and it can detect EVs using only 5 µL of ascite fluid from tumor patients in 15 min. While this approach does not rely on the conventional SPR-based detection setup, the utilization of hydrogel metamaterials holds promise for advancing optical EV sensing, especially for POC applications.

The sensitivity of SPR-based detection is usually higher than that of LSPR-based detection; however, it is less convenient to miniaturize SPR-based setups. In order to achieve the full potential of SPR-based detection for advanced healthcare, there is a need to miniaturize the bulky instruments required for the coupling of light with propagating SPPs and spectrometers [92]. Also, it is encouraging to achieve advancements in nanofabrication and innovative manufacturing methods, including flexible substrates, 3D printing, and fiber optics for making SPR-based sensors [93–95].

## Surface-enhanced Raman spectroscopy (SERS)-based EV sensing

**Fig. 4:**
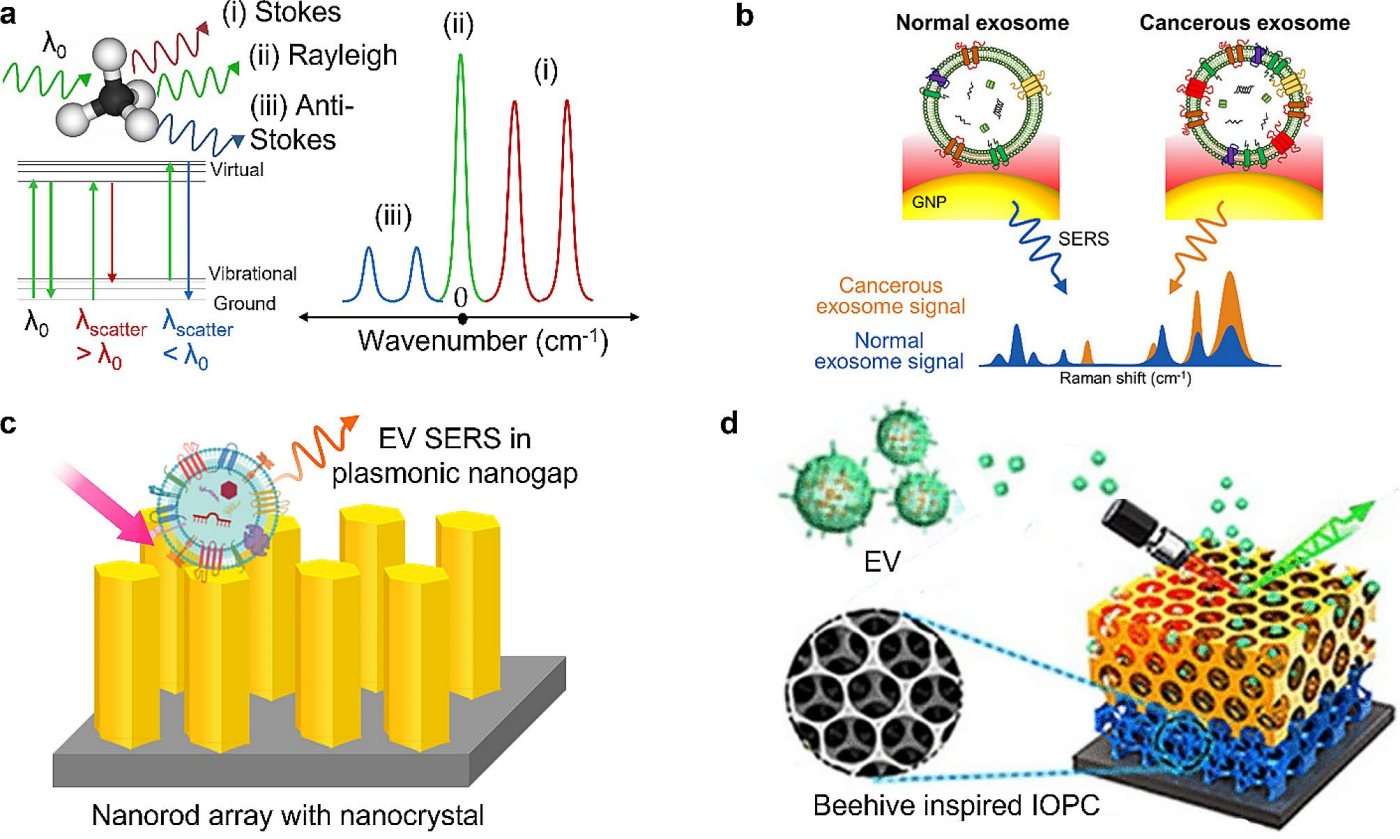
Surface-enhanced Raman spectroscopy for EV sensing. **a**, Principle of Raman spectroscopy and generation of Stokes and Anti-Stokes signals by a vibrating molecule. **b**, SERS for distinguishing normal and cancer EVs. Reprinted with permission [101, 102] Copyright 2018 American Chemical Society. **c**, Plasmonic nanogaps for EV SERS-based sensing using nanorod array [103]. **d**, Beehive-inspired inverse opal photonic crystal (IOPC) with plasmonic coating for EV SERS. Reprinted with permission [104] Copyright 2018 American Chemical Society

Each molecule has specific vibrational energy levels, and Raman spectroscopy offers a precise means of discerning transitions between them. Raman spectroscopy measures the inelastic scattering of photons upon the excitation of molecules to a virtual state by incident light. This inelastic scattering includes both Stokes and anti-Stokes phenomena, leading to energy loss or gain of scattered photons, respectively (see Fig. 4a left panel, red and blue arrow lines). Elastic (Rayleigh) scattering, typically observed as a dominant peak at 0 cm^− 1^ on Raman shift (Fig. 4a right panel, green line), involves the absorption and re-emission of photons without altering their energy state (Fig. 4a left panel, green arrow line).

Since its discovery, Raman spectroscopy has maintained its status as a versatile and non-invasive molecular fingerprinting technique [96]. This is because the Raman spectrum provides insights into molecular structures that can be extracted from the vibrational energies. However, as the Raman shift relies on the change in polarizability of the electron cloud in a molecule with very low probability and optical cross-section, the Raman signal is insufficient for practical application. Meanwhile, the Raman signal of a molecule can be amplified by a factor of 10^5^–10^6^ orders of magnitude when the molecule is adsorbed on a plasmonic nanoparticle or substrate, known as surface-enhanced Raman spectroscopy (SERS) [97–100].

SERS can be attributed to two principal enhancement mechanisms: electromagnetic and chemical enhancement [105]. The electromagnetic enhancement involves two contributing factors, one related to the enhanced incident energy by LSPR, accompanying improved excitation of molecule’s Raman modes, expressed as ${\left|{E}_{loc}/{E}_{0}\right|}^{2}$, while the other contributes to the enhancement of outgoing Raman signal, also with the factor of ${\left|{E}_{loc}/{E}_{0}\right|}^{2}$. This is why the overall enhancement factor is expressed as ${\left|{E}_{loc}/{E}_{0}\right|}^{4}$. Chemical enhancement is due to changes in molecular electronic distribution and the formation of new charge transfer states, resulting in an additional enhancement of 1–3 orders of magnitude [99, 106]. The electromagnetic enhancement is most significant when the LSPR peak falls within the wavelength range of the incident laser and Raman scattering [100].

While SERS has traditionally been considered as laboratory equipment, recent significant advancements have propelled it towards portable technologies, including portable spectrometers, on-chip sample preparations [107], and optical trapping of single vesicles [108]. The versatility in identifying molecules based on their unique vibrational fingerprints can offer the potential for high-throughput detection of unknown EV biomarkers. One of the key strengths of SERS lies in its ability to distinguish the heterogeneity of EVs. Depending on their origin, EVs exhibit mixed and complex Raman spectra, providing valuable information such as cell lineage, biofluid source, and the composition of their contents, including proteins, lipids, and other molecules. While analysis of individual peaks is challenging, specific peaks can serve as signatures of diseases. For example, a peak at 1087 cm^− 1^ distinguishes several cancer cell-derived EVs from their normal counterparts [104]. From 93 human subjects, SERS successfully classified 45 prostate cancer patients, 8 colon cancer patients, 15 lung cancer patients, 15 liver cancer patients and 10 normal individuals. The distinctive peak at 1087 cm^− 1^arises from the phosphate group, associated with a protein phosphorylation process commonly linked to cancer. These significantly distinct SERS profiles between normal and cancerous EVs (Fig. 4b) result from the superimposition of varied Raman fingerprints of protein markers [101]. Due to the heterogeneous peak compositions, statistical tools such as principal component analysis (PCA) are useful in identifying significant patterns and informative correlations from SERS data rather than focusing on a single molecule’s Raman signature [102, 109, 110]. This approach detected concentrations as low as 580 fM of EVs from cell culture fluid using SERS [109]. In another work on cancer EVs (NSCLC), the extracted SERS signatures exhibited a 90% correlation with the number of cancer EVs present [102]. Similarly, employing SERS combined with PCA on lung cancer EVs, with a concentration as low as 10^9^ vesicles/ml, resulted in classification with 95.3% sensitivity and 97.3% specificity [110]. The plasmonic substrates can be designed to capture EVs or bacterial MVs and for SERS sensing effectively. To enhance Raman signals, target molecules need to be near the nanoplasmonic structure, essentially within the hotspots of the electric field. One approach involves using van der Waals interaction, facilitating the physical entrapment of the vesicles near the plasmonic nanostructure, thereby generating SERS signal [109]. Another intriguing structure is a vertical array of gold nanorods [103] with better LSPR coupling than a variety of GNPs (Fig. 4c). The SERS enhancement factor (EF) can be in the range of 10^8^. Also, macroporous inverse opals (IO), inspired by the structure of a beehive made of metal oxides such as silica and titania with embedded plasmonic surfaces, present advanced sensing ability. These IO structures exhibit the remarkable ability to tune and concentrate visible light by changing the pore size (Fig. 4d) [104]. The synthesis of these inverse opals is straightforward, relying on a wet chemical approach that yields impressively regular porous structure with high surface area and photonic crystal properties. An EF in the range of 10^5^ is demonstrated. Owing to the similarity in size between the EVs and IO’s pore dimensions, they can be effectively entrapped and isolated in the pores. This enables the efficient measurement of SERS signals. The synergistic effect from plasmonic and photonic crystal coupling contributes to efficient EV detection for classifying various cancers.

Raman spectroscopic investigation can reveal several interactions in microbiology, such as molecular exchanges and communications from a cell to its environment and between cells, thereby assessing a microbial ecosystem [111]. It is attractive that the possible scale of the microbe system under Raman spectroscopic investigation can be as small as the human gut microbiome or as big as the oceanic environment. SERS can significantly enhance the sensitivity of Raman spectroscopy of bacterial MVs in a microbiome. Raman spectroscopic characterization of bacterial MVs that promote plant health, is shown to reveal the effect of outer stressors on the OMV contents and composition, thus helping to monitor the environmental conditions and environmental impact on the plant [112]. Similarly, bacterial MVs produced by human pathogens can also be monitored by Raman spectroscopic analysis [113]. Combined with machine learning- based classification, it is possible to identify antibiotic- resistant strains. In-situ Raman spectroscopy can be used to inspect human gut microbes, identifying the presence of specific antibiotic-resistant bacteria related to the person’s antibiotic intake history [114]. Gut bacterial MVs can be indicative biomarkers of IgA secretion related to immunity [115]. It is also shown that gut bacterial MVs can be explicitly involved in human immunity against viruses, indicating the side effects of antibiotic medicines [116]. Bacterial MVs have excellent prospects in human disease and environmental monitoring, and SERS can offer enhanced sensing and molecular fingerprinting of MVs. Combining the amplified sensitivity of SERS with promising work in the miniaturization of spectroscopy instruments [117] paves the way for precision medicine in clinical settings. It can deeply explore questions such as the impact of gut bacteria on human diseases.

In some cases, SERS spectra obtained from EVs or bacterial MVs of the exact cell origin might exhibit a change in peaks due to the non-uniform enhancement of SERS based on the proximity of the EV to the plasmonic substrate [118]. Although this can be addressed by employing substrates with uniform EM field, suitable statistical tools such as multivariate curve resolution-alternating least squares can solve most additive mixing problems in SERS signal analysis without the need for precise reference spectra.

## Summary, challenges, and future directions

In the past few years, nanoplasmonic optical sensors, owing to their intense light concentration and label-free sensing capabilities, have significantly advanced toward minimally invasive clinical applications and precision diagnostics of diseases. As discussed in this Review, nanoplasmonic EV or bacterial MV sensing techniques have extended sensitivity, throughput, and selectivity limits in colorimetry, refractometric sensing, Raman spectroscopy, and mass spectrometry. However, several challenges in on-chip sample preparation, miniaturization, and multiplexing must be addressed to move toward point-of-care diagnostics. While engineered nanoplasmonics such as nanogaps, nanocavity, nanoholes, multimers, and thin films with enhanced optical properties provide extreme electric field enhancement, it is essential to focus on integrating them into multiplexed, high-throughput sensing for developing next-generation devices. Furthermore, providing a proper sensing platform for POC systems such as low-cost plasmonic substrates, smartphone-based sensing, and data/AI-driven analysis schemes is necessary. Bottom-up nanopatterning for nanoplasmonic substrate fabrication can be an alternative to expensive lithography [119].

The following points outline essential directions for future research: (1) Samples-to-answer chips can significantly reduce the gap from the research laboratory to patient care. The development of on-chip detection of EVs is a significant area as a POC device in personalized healthcare solutions. Bacterial MV detection is a developing field of research and provides a powerful tool for environmental monitoring and disease detection where a POC system will significantly impact. Though complete on-chip sample preparation remains to be achieved, sensor technologies that do not require separate biological processing or spectrometers are up-and-coming, particularly suitable in resource-limited settings; (2) Rapid EV isolation is often the rate-limiting task in EV research. Integrating EV isolation with sensor chips remains essential for commercializing EV-based early disease diagnosis technology, especially for at-home use; (3) Advancements in sensor throughput and multiplexing capabilities beyond individual patients can enhance the scope of commercial applications and significantly accelerate clinical research. For example, there is considerable interest in simplifying the SPR imaging machinery to the level of dark-field imaging using a smartphone [120], which can be efficiently combined with machine learning techniques to develop next-generation nanoplasmonic sensors; (4) Improving portability of spectrometers [117] and miniaturization of the instruments are the key to advanced POC EV/MV sensing using versatile molecular fingerprinting techniques such as SERS.

Furthermore, in addition to serving as critical biomarkers for early disease diagnosis, EVs and bacterial MVs hold promising applications in cancer and disease prognosis and therapy. The growing demand for commercially produced EVs indicates the need for rapid, reusable EV sensors to ensure quality control during manufacturing [121]. Developing a minimally invasive and non-destructive EV sensor without bio-toxic dye-based sensing elements is promising for therapeutic applications.

Recent advancements in extreme scattering high-Q cavities [122], graphene plasmonics [123], and metamaterial plasmonics [124] show the exciting and continuing evolution of improving detection sensitivity. Although translating the full potential of nanoplasmonics into a biosensor device for EV or bacterial MV analysis presents challenges, current research offers promise. Future generations of nanoplasmonic biosensors are expected to address these challenges, enabling minimally invasive clinical applications and precise POC diagnostics of various diseases.

## Abbreviations

EV: Extracellular vesicle
MV: Membrane vesicle
SERS: Surface-enhanced Raman spectroscopy
ELISA: Enzyme-linked immune-assay
mRNA: messenger RNA
miRNA: microRNA
circRNA: circular RNA
POC: Point-of-care
RI: Refractive index
LSPR: Localized surface plasmon resonance
*S*: Sensitivity
*E*_*b*_: Efficiency of binding of analytes
*c*: concentration
*P*: Output signal
*E*: Electric field
EF: Enhancement factor
NP: Nanoparticle
GFP: Green fluorescent protein
LFIA: Lateral flow immunoassay
GNP: Gold nanoparticle
MS: Mass spectrometry
NSCLC: Non-small cell lung cancer
PRET: Plasmonic resonance energy transfer
rPRET: Reversed plasmonic resonance energy transfer
QBET: Quantum biological electron tunneling
MDV: Mitochondria-derived vesicle
BHQ–3: Black hole quencher
AzoR: Azoreductase
Cyt P450: Cytochrome P450
Cyt C: Cytochrome C
SPP: Surface plasmon polariton
RU: Resonance unit
EOT: Extraordinary optical transmission
GF: Gold film
CP: Critical point
PCA: Principal component analysis
IO: Inverse opal
